# Prediction of Drugs Target Groups Based on ChEBI Ontology

**DOI:** 10.1155/2013/132724

**Published:** 2013-11-20

**Authors:** Yu-Fei Gao, Lei Chen, Guo-Hua Huang, Tao Zhang, Kai-Yan Feng, Hai-Peng Li, Yang Jiang

**Affiliations:** ^1^Department of Surgery, China-Japan Union Hospital of Jilin University, Changchun 130033, China; ^2^College of Information Engineering, Shanghai Maritime University, Shanghai 201306, China; ^3^Institute of Systems Biology, Shanghai University, Shanghai 200444, China; ^4^Beijing Genomics Institute, Shenzhen Beishan Industrial Zone, Shenzhen 518083, China; ^5^CAS-MPG Partner Institute for Computational Biology, Shanghai Institutes for Biological Sciences, Chinese Academy of Sciences, Shanghai 200031, China

## Abstract

Most drugs have beneficial as well as adverse effects and exert their biological functions by adjusting and altering the functions of their target proteins. Thus, knowledge of drugs target proteins is essential for the improvement of therapeutic effects and mitigation of undesirable side effects. In the study, we proposed a novel prediction method based on drug/compound ontology information extracted from ChEBI to identify drugs target groups from which the kind of functions of a drug may be deduced. By collecting data in KEGG, a benchmark dataset consisting of 876 drugs, categorized into four target groups, was constructed. To evaluate the method more thoroughly, the benchmark dataset was divided into a training dataset and an independent test dataset. It is observed by jackknife test that the overall prediction accuracy on the training dataset was 83.12%, while it was 87.50% on the test dataset—the predictor exhibited an excellent generalization. The good performance of the method indicates that the ontology information of the drugs contains rich information about their target groups, and the study may become an inspiration to solve the problems of this sort and bridge the gap between ChEBI ontology and drugs target groups.

## 1. Introduction

Identification of target proteins of drugs is of importance in the drug discovery pipeline [1] because drugs exert their functions by hitting some proteins, that is, their target proteins, in human tissues. On the other hand, in addition to their therapeutic effects, most of the drugs have some undesirable side effects caused also by hitting some target proteins. If a drug with unclear undesirable side effects was brought into the market, it is a potential hazard to both pharmaceutical companies and their consumers. Thus, studying the target proteins of a drug is highly beneficial to the treatment of diseases and reduction of side effects. However, identification of drugs target proteins by experiments needs lots of time and money. It is necessary to establish effective computational methods to tackle this problem which can provide useful references. 

Many efforts have been made to identify drugs target proteins in the past few years, such as docking simulations [2, 3], literature text mining [4], combination of chemical structure and protein structural information or functional information [5–8], side effect similarity [9], and so forth. In this paper, we attempted a novel method using the ontology information of compounds, which was similar to gene ontology of proteins, to identify drugs target proteins. With the discovery of novel candidate drugs, the quantity of all candidate pairs of drugs and target proteins is tremendously large, preventing researchers to carry out an exhaustive search of drugs target proteins. In view of this, a necessary step is to establish an effective method to reduce the candidate proteins for each query drug, that is, reducing the search space by deducing the kind of functions a drug may have. According to the data in KEGG (Kyoto Encyclopedia of Genes and Genomes, http://www.genome.jp/kegg/) [10], the target proteins of drugs could be divided into the following five groups: (1) G Protein-coupled Receptors, (2) Cytokine Receptors, (3) Nuclear Receptors, (4) Ion Channels, and (5) Enzymes. If one can establish a method to correctly predict the target groups of a query drug, the possible target proteins would be limited only to the predicted group, facilitating further analyses.

In the past few years, many novel compounds have been discovered with the advance of combinatorial chemistry. To record these compounds, some online databases are established, such as KEGG [10], STITCH (Search Tool for Interactions of Chemicals) [11], and ChEBI (Chemical Entities of Biological Interest) [12], from which users can retrieve all sorts of information about the compounds, for example, their structures, activities, reactions, and so on. Furthermore, their information can also be used to infer the attributes of novel compounds [5, 7, 8, 13–15]. In the paper, we employed compound ontology information, named as ChEBI ontology, to infer the target group of a novel drug, that is, a predictor that was built to predict the target group of drugs based on ChEBI ontology. A benchmark dataset consisting of 876 drugs was established by collecting data in KEGG, from which a training dataset and a test dataset were obtained by splitting the data. Jackknife test demonstrates an overall prediction accuracy of 83.12% and independent test achieves a prediction accuracy of 87.50%, indicating that the predictor has excellent generalization. We hope that the predictor may facilitate the discovery of new therapeutic or undesirable effects of existing drugs.

## 2. Materials and Methods

### 2.1. Dataset

2,795 drugs were retrieved from Chen and Zeng's study [8], which were downloaded from KEGG (http://www.genome.jp/kegg/) [10]. According to their target proteins, these drugs were classified into the following five groups: (1) G Protein-coupled Receptors, (2) Cytokine Receptors, (3) Nuclear Receptors, (4) Ion Channels, and (5) Enzymes. We then screened the data with the following rules: drugs without ChEBI ontology information were excluded, resulting in 895 drugs; drugs belonging to more than one group were excluded, resulting in 879 drugs; and because there were only 3 drugs in Cytokine Receptors—not enough to build an effective prediction model on the group, these drugs and the group were also excluded. Thus, we obtained a benchmark dataset **S** containing 876 drugs allocated into four groups. The distribution of these drugs is listed in column 5 of Table 1.  The codes of the drugs in each group are available in Supplementary Material I available online at http://dx.doi.org/10.1155/2013/132724. 

To evaluate the generalization of the predictor, the benchmark dataset **S** was divided into a training dataset **S**_tr⁡_ and a test dataset **S**_te_, where **S**_te_ was constructed by randomly selecting 88 (10%) drugs in **S** and the rest in **S** comprised **S**_tr⁡_. The number of drugs in each group in the training and test dataset was listed in columns 3 and 4 of Table 1, respectively.

### 2.2. Prediction Based on ChEBI Ontology

The term “ontology” derived from philosophy, meaning the theory or study of the basic characteristics of all reality. Since gene ontology, the established ontology information about proteins, is deemed as a very useful tool for investigating various attributes of proteins [16–21], similarly, the ontology information of compounds may also facilitate the study of various attributes of compounds. 

ChEBI, a well-known compound database, contained some important ontology information about compounds named as ChEBI ontology [12]. It consists of four subontologies: (1) Molecular Structure, (2) Biological Role, (3) Application, and (4) Subatomic Particle, which may be suitable for the prediction of various attributes of compounds. The information of ChEBI ontology was retrieved from ftp://ftp.ebi.ac.uk/pub/databases/chebi/ontology/ (“chebi.obo”, July 2012). Ontologies are controlled vocabularies which can be conceived as graph-theoretical structures consisting of “terms” forming the node set and “relations” of two terms forming the edge set [22]. Based on the “terms” and “relations” (including “is_a” and “relationship”) in the obtained file, a graph with 31,813 nodes and 64,514 edges was established. As for the two terms, the smaller the distance is between them, the more intimate the “relations” are implicated between them. Thus, the distance of terms *t* and *t*′, denoted by *d* (*t*, *t*′), would be used to measure the relationship of compounds.

For two compounds *c*_1_ and *c*_2_, *T*(*c*_1_) and *T*(*c*_2_) are an ontology term set of *c*_1_ and *c*_2_, respectively. The following formula was used to measure the functional relationship of *c*_1_ and *c*_2_:

(1) $$\begin{array}{c} Q\left(c_{1},c_{2}\right)=\text{Mean}\left\{d\left(t_{1},t_{2}\right):t_{1}\in T\left(c_{1}\right),t_{2}\in T\left(c_{2}\right)\right\}. \end{array}$$

The smaller the *Q*(*c*_1_, *c*_2_) is, the stronger the functional relationship would be shared by *c*_1_ and *c*_2_.

For a query drug *d*_*q*_, its target group was predicted according to the following steps.

(i) Find drugs in the training set **S**′, say, *d*_1_, *d*_2_,…, *d*_*s*_, such that
  (2) Q(dq,d1)=Q(dq,d2)=⋯=Q(dq,ds)=min⁡d∈S′ Q(dq,d).
(ii) The target groups of *d*_1_, *d*_2_,…, *d*_*s*_ were put into a voting system.
(iii) The target group with the most votes is deemed to be the predicted target group of *d*_*q*_. Note that if more than one target group is receiving the most votes, randomly select one of them as the predicted result.

### 2.3. Prediction Based on Chemical Interaction

In recent years, the idea of “systems biology” is penetrating into the prediction of various attributes of proteins and compounds and is considered to be very useful [13, 14, 23–25]. The constructed methods were all based on the fact that interactive proteins and compounds often share common features. To define the interactive compounds, we downloaded the chemical interaction files from STITCH ((chemical_chemical.links.detailed.v3.1.tsv.gz) http://stitch.embl.de/download/chemical_chemical.links.detailed.v3.1.tsv.gz, http://stitch.embl.de/) [11], a well-known database including the interaction information of proteins and chemicals. In the obtained file, each interaction is composed of two chemicals and five kinds of scores. In detail, the first four kinds of scores are estimated according to the structures, activities, reactions, and cooccurrence in the literature of two chemicals [11], while the last kind of score is calculated by integrating the aforementioned four kinds of scores. It is reasonable to use the last kind of score to indicate the interactivity of two chemicals. Thus, it was adopted here to indicate the interactivity of two chemicals; that is, two chemicals are interactive chemicals if and only if the last kind of score of the interaction between them is greater than 0. For the later formulation, we denote the score of chemicals *c*_1_ and *c*_2_ by *I*(*c*_1_, *c*_2_). In particular, if *c*_1_ and *c*_2_ are noninteractive chemicals, we set *I*(*c*_1_, *c*_2_) = 0.

As described above, the interactive compounds share common features with higher possibility than noninteractive ones. In view of this, the target group of a query drug *d*_*q*_ can be determined by its interactive compounds in the training set. The detailed procedure of the method is almost similar to that of the method in Section 2.2. Now, instead of (2), we used the following formula to select drugs in the first step

(3) I(dq,d1)=I(dq,d2)=⋯=I(dq,ds)=max⁡d∈S′ I(dq,d).

### 2.4. Jackknife Test

In statistical prediction, there are three cross-validation methods: independent dataset test, subsampling (or *k*-fold crossover) test, and jackknife test [13], which are often used to evaluate the performance of various classifiers. Among them, jackknife test is deemed the least arbitrary [13] because the test sample and training samples are always open. Furthermore, the classifier evaluated by jackknife test can always provide a unique result for a given dataset. Accordingly, it has been widely used to examine the performance of various classifiers in recent years [13, 26–36]. Here, we also adopted it to evaluate the current method.

## 3. Results and Discussions

As described in Section 2.1, the benchmark dataset **S** was divided into two datasets, **S**_tr⁡_ and **S**_te_, consisting of 788 and 88 drugs, respectively. The method based on ChEBI ontology was applied to predict the target groups of drugs in these two datasets. The detailed results were given in the following sections.

### 3.1. Performance of the Predictor on the Training Dataset

As for the 788 drugs in the training dataset **S**_tr⁡_, the predictor based on ChEBI ontology was evaluated by jackknife test. The prediction results were listed in column 2 of Table 2, from which we can see that the prediction accuracies for each target group were 93.38%, 73.17%, 60.55%, and 84.62%, respectively, while the overall prediction accuracy was 83.12%. Since there are four target groups investigated by the study, the average correct rate would be 25% if one identifies drugs target groups in **S**_tr⁡_ by random guesses, which is much lower than the overall prediction accuracy obtained by our method. Compared to the results in Chen and Zeng's work [8], in which a similarity-based method was proposed to predict drugs target groups, our results are also very competitive because the prediction accuracies in their work were less than 80%. All of these suggest that the proposed predictor performs fairly well on the training dataset.

### 3.2. Performance of the Predictor on the Test Dataset

As for the 88 drugs in the test dataset **S**_te_, the predictor was modeled only based on the training dataset **S**_tr⁡_ without involving **S**_te_. The prediction accuracies for each group and the overall accuracy were listed in column 3 of Table 2. It can be seen that the prediction accuracies for each group were 100%, 69.23%, 55.56%, and 90.32%, respectively, while the overall prediction accuracy was 87.50%, which is even better than that of the training dataset, indicating that the predictor has an excellent generalization. 

### 3.3. Comparison of the Predictors Based on ChEBI Ontology and Chemical Interaction

The method based on chemical interaction described in Section 2.3 is popular for predicting various attributes of compounds [13, 14]. Thus, we compared the performances of these two methods in identifying drugs target groups as follows.

To compare the methods with the same datasets, all samples in the benchmark dataset **S** were used to make prediction; that is, two predictors were conducted to predict the target groups of samples in **S** evaluated by jackknife test. The prediction results obtained by these two methods were listed in Table 3. It is observed that the overall prediction accuracy for the predictor using ChEBI ontology was 84.70%, which is a little higher than that of the method using chemical interaction. In detail, the prediction accuracy for the target group “G Protein-coupled Receptors” obtained by the proposed method was much higher than the corresponding accuracy obtained by the method based on chemical interaction, the prediction accuracies for the target group “Enzymes” obtained by these two methods were almost the same, while the prediction accuracies for the rest two target groups obtained by the proposed method were lower than those obtained by the method based on chemical interaction. All of these indicate that the two predictors perform at the same level on the benchmark dataset **S**. Thus, it can be inferred that strong links may exist between ChEBI ontology and chemical interactions. 

### 3.4. Analysis of the Relationship of Drugs Ontology Information and Their Target Group

From Sections 3.1–3.3, the ChEBI ontology information of compounds connects strongly with their targets' information. In this section, some examples are picked up to confirm this and to reinforce the understanding of using ChEBI to categorize drugs into their target groups. 

The drug “D00146” is a sample in the training dataset **S**_tr⁡_. Its target group is “G Protein-coupled Receptors” and it hits the ontology term “CHEBI:3892.” According to the procedure of the method based on ChEBI ontology, 13 drugs in **S**_tr⁡_ (listed in Table 4) were found, satisfying the function *Q*(•) to be minimum. It is observed that 11 out of 13 drugs are in the target group “G Protein-coupled Receptors” and the rest two drugs are in the target group “Enzymes.” Thus, the target group “G Protein-coupled Receptors” got 11 votes, “Enzymes” got 2 votes, and the rest target groups did not get any votes. Accordingly, the target group of “D00146” is predicted to be “G Protein-coupled Receptors,” which is indeed its true target group. Another example is the drug “D00387” in the test dataset **S**_te_, which is in the target group “Ion Channels.” According to its ontology term “CHEBI:9674,” we found 20 drugs in **S**_tr⁡_, such that the function *Q*(•) achieved a minimum. These 20 drugs were listed in Table 5, from which we can see that 12 drugs are in target group “Ion Channels” and 8 drugs are in target group “G Protein-coupled Receptors.” Thus, the result of “D00387” is predicted to be in the target group “Ion Channels.” It is also predicted correctly. 

The two examples in the above paragraph show that the target information of these drugs is indeed related to their ontology information. The good performance of the predictor demonstrated the validity of using ontology information to predict drugs target groups. 

## 4. Conclusion

This study employed ChEBI ontology to categorize drugs based on their target proteins. The good performance of the method suggests that ontologies are good indicators of drugs target groups. However, only about 30% of the samples reported in KEGG were investigated in this study due to the lack of ontology information of most drugs. It is anticipated that the method would be more effective at the prediction with the development of ChEBI ontology and hopefully a multilabel classifier may be developed to allocate some drugs to more than one category in the near future. 

## Supplementary Material

Supplementary Material I lists 876 drug samples investigated in this study.

## Figures and Tables

**Table 1:** The number of drugs in each group.

Index	Target group	Number of drugs		
		Training dataset	Test dataset	Total
1	G Protein-coupled Receptors	272	35	307
2	Nuclear Receptors	82	13	95
3	Ion Channels	109	9	118
4	Enzymes	325	31	356

—	Total	788	88	876

**Table 2:** The correct prediction rates of the method based on ChEBI ontology.

Target group	Training dataset	Test dataset
G Protein-coupled Receptors	93.38%	100%
Nuclear Receptors	73.17%	69.23%
Ion Channels	60.55%	55.56%
Enzymes	84.62%	90.32%
Overall	83.12%	87.50%

**Table 3:** The prediction accuracies of the method based on ChEBI ontology and chemical interaction on the benchmark dataset evaluated by jackknife test.

Target group	Prediction based on ChEBI ontology	Prediction based on chemical interaction
G Protein-coupled Receptors	94.46%	83.71%
Nuclear Receptors	76.84%	82.11%
Ion Channels	61.02%	77.97%
Enzymes	86.24%	87.08%
Overall	84.70%	84.13%

**Table 4:** The 13 drugs which are closest to “D00146” in **S**_tr⁡_.

Drug	Target group	Ontology term
D00089	G Protein-coupled Receptors	CHEBI:7872
D00101	G Protein-coupled Receptors	CHEBI:9937
D00176	G Protein-coupled Receptors	CHEBI:35940
D00284	G Protein-coupled Receptors	CHEBI:3901
D00291	G Protein-coupled Receptors	CHEBI:4450
D00410	Enzymes	CHEBI:44241
D00994	Enzymes	CHEBI:4759
D01002	G Protein-coupled Receptors	CHEBI:4025
D01163	G Protein-coupled Receptors	CHEBI:31554
D02783	G Protein-coupled Receptors	CHEBI:337298
D07431	G Protein-coupled Receptors	CHEBI:64628
D07759	G Protein-coupled Receptors	CHEBI:4024
D07905	G Protein-coupled Receptors	CHEBI:4822

**Table 5:** The 20 drugs which are closest to “D00387” in **S**_tr⁡_.

Drug	Target group	Ontology term	Drug	Target group	Ontology term
D00225	Ion Channels	CHEBI:2611	D00293	Ion Channels	CHEBI:49575
D00300	G Protein-coupled Receptors	CHEBI:4636	D00311	Ion Channels	CHEBI:4858
D00430	Ion Channels	CHEBI:9073	D00494	G Protein-coupled Receptors	CHEBI:8461
D00506	Ion Channels	CHEBI:8069	D00549	Ion Channels	CHEBI:44915
D00669	G Protein-coupled Receptors	CHEBI:4637	D01177	G Protein-coupled Receptors	CHEBI:32091
D01205	G Protein-coupled Receptors	CHEBI:31472	D01310	Ion Channels	CHEBI:32124
D01372	Ion Channels	CHEBI:32315	D01485	G Protein-coupled Receptors	CHEBI:31981
D01657	Ion Channels	CHEBI:52993	D02419	G Protein-coupled Receptors	CHEBI:34720
D02624	Ion Channels	CHEBI:53760	D08283	Ion Channels	CHEBI:111762
D08473	G Protein-coupled Receptors	CHEBI:8802	D08690	Ion Channels	CHEBI:10125
