# Supplementary material for: Prediction of Drugs Target Groups Based on ChEBI Ontology
Source: Biomed Res Int. 2013 Nov 20;2013:132724. doi: 10.1155/2013/132724 (PMC3853244; doi:10.1155/2013/132724)
Supplement: Supplementary file 1 — Supplementary Material I lists 876 drug samples investigated in this study. [file 132724.f1.pdf]

**Supplementary Material I.** The benchmark dataset consisted of 876 drugs, which were classified into 4 target groups.

## 1. Drugs in target group "G Protein-coupled Receptors"

| | | | | | |
|---|---|---|---|---|---|
| D00059 | D00076 | D00079 | D00089 | D00095 | D00101 |
| D00113 | D00116 | D00136 | D00138 | D00146 | D00147 |
| D00176 | D00180 | D00232 | D00234 | D00235 | D00241 |
| D00255 | D00270 | D00274 | D00281 | D00283 | D00284 |
| D00291 | D00295 | D00300 | D00308 | D00318 | D00320 |
| D00356 | D00373 | D00378 | D00390 | D00400 | D00411 |
| D00415 | D00419 | D00422 | D00426 | D00432 | D00451 |
| D00454 | D00458 | D00465 | D00479 | D00480 | D00482 |
| D00483 | D00485 | D00493 | D00494 | D00502 | D00503 |
| D00511 | D00513 | D00514 | D00520 | D00522 | D00523 |
| D00524 | D00559 | D00560 | D00561 | D00563 | D00597 |
| D00598 | D00599 | D00603 | D00608 | D00609 | D00610 |
| D00627 | D00632 | D00634 | D00644 | D00646 | D00659 |
| D00662 | D00663 | D00666 | D00669 | D00672 | D00674 |
| D00676 | D00682 | D00684 | D00702 | D00715 | D00717 |
| D00722 | D00723 | D00726 | D00769 | D00774 | D00778 |
| D00779 | D00780 | D00782 | D00789 | D00794 | D00797 |
| D00798 | D00799 | D00800 | D00819 | D00836 | D00837 |
| D00838 | D00839 | D00842 | D00843 | D00847 | D00987 |
| D00989 | D00996 | D01000 | D01002 | D01008 | D01017 |
| D01019 | D01024 | D01025 | D01028 | D01044 | D01051 |
| D01096 | D01118 | D01148 | D01163 | D01164 | D01172 |
| D01177 | D01182 | D01205 | D01226 | D01227 | D01236 |
| D01295 | D01297 | D01307 | D01321 | D01336 | D01358 |
| D01360 | D01369 | D01373 | D01399 | D01428 | D01448 |
| D01452 | D01462 | D01468 | D01471 | D01485 | D01685 |
| D01692 | D01699 | D01745 | D01871 | D01939 | D01964 |
| D01973 | D02004 | D02022 | D02037 | D02070 | D02071 |
| D02076 | D02081 | D02082 | D02100 | D02116 | D02147 |
| D02211 | D02212 | D02213 | D02234 | D02235 | D02249 |
| D02271 | D02338 | D02340 | D02342 | D02343 | D02349 |
| D02357 | D02358 | D02363 | D02369 | D02374 | D02419 |
| D02599 | D02613 | D02721 | D02724 | D02730 | D02738 |
| D02824 | D02825 | D02829 | D02846 | D02968 | D02976 |
| D02983 | D03165 | D03197 | D03503 | D03504 | D03505 |
| D03535 | D03556 | D03621 | D03622 | D03654 | D03693 |
| D03704 | D03713 | D03729 | D03879 | D04006 | D04040 |
| D04057 | D04157 | D04187 | D04716 | D04883 | D04970 |
| D05008 | D05113 | D05127 | D05312 | D05523 | D05575 |

| | | | | | |
|---|---|---|---|---|---|
| D05632 | D05738 | D05792 | D05938 | D06056 | D06213 |
| D06247 | D06330 | D07122 | D07124 | D07129 | D07132 |
| D07156 | D07218 | D07229 | D07312 | D07377 | D07398 |
| D07431 | D07461 | D07463 | D07482 | D07483 | D07489 |
| D07511 | D07526 | D07534 | D07538 | D07543 | D07593 |
| D07617 | D07620 | D07624 | D07662 | D07665 | D07667 |
| D07713 | D07748 | D07759 | D07765 | D07803 | D07805 |
| D07809 | D07820 | D07837 | D07838 | D07862 | D07868 |
| D07870 | D07874 | D07878 | D07887 | D07890 | D07900 |
| D07905 | D07906 | D07916 | D07977 | D07990 | D08090 |
| D08113 | D08146 | D08305 | D08339 | D08397 | D08426 |
| D08473 | D08585 | D08600 | D08611 | D08636 | D08685 |
| D08687 | | | | | |

**2. Drugs in target group "Nuclear Receptors"**

| | | | | | |
|---|---|---|---|---|---|
| D00066 | D00067 | D00075 | D00088 | D00129 | D00165 |
| D00182 | D00185 | D00187 | D00244 | D00246 | D00279 |
| D00289 | D00292 | D00299 | D00312 | D00316 | D00327 |
| D00334 | D00395 | D00407 | D00408 | D00443 | D00462 |
| D00565 | D00577 | D00585 | D00586 | D00596 | D00689 |
| D00898 | D00950 | D00951 | D00952 | D00954 | D00956 |
| D00957 | D00958 | D00961 | D00962 | D00965 | D00966 |
| D00972 | D00975 | D00977 | D01011 | D01112 | D01115 |
| D01161 | D01217 | D01272 | D01294 | D01327 | D01357 |
| D01366 | D01368 | D01387 | D01402 | D01442 | D01464 |
| D01510 | D01619 | D01637 | D01639 | D01689 | D01708 |
| D01820 | D02217 | D02287 | D02367 | D02815 | D03106 |
| D03493 | D03521 | D03595 | D03696 | D03697 | D03917 |
| D04104 | D04208 | D05209 | D06171 | D06551 | D07116 |
| D07495 | D07536 | D07715 | D07719 | D07726 | D07827 |
| D07939 | D08052 | D08166 | D08167 | D08610 | |

**3. Drugs in target group "Ion Channels"**

| | | | | | |
|---|---|---|---|---|---|
| D00011 | D00110 | D00225 | D00228 | D00250 | D00252 |
| D00267 | D00271 | D00280 | D00293 | D00294 | D00303 |
| D00311 | D00319 | D00326 | D00331 | D00336 | D00354 |
| D00358 | D00380 | D00387 | D00394 | D00418 | D00430 |
| D00437 | D00438 | D00477 | D00492 | D00506 | D00512 |
| D00533 | D00537 | D00538 | D00539 | D00549 | D00551 |
| D00552 | D00553 | D00615 | D00616 | D00619 | D00631 |
| D00639 | D00640 | D00647 | D00649 | D00693 | D00694 |
| D00701 | D00706 | D00711 | D00714 | D00732 | D00733 |
| D00735 | D00738 | D00758 | D00759 | D00760 | D00765 |
| D00766 | D00767 | D00811 | D00812 | D00815 | D00821 |

| | | | | | |
|---|---|---|---|---|---|
| D00822 | D00825 | D01173 | D01179 | D01253 | D01285 |
| D01287 | D01310 | D01354 | D01372 | D01450 | D01546 |
| D01599 | D01657 | D02074 | D02078 | D02086 | D02087 |
| D02182 | D02220 | D02356 | D02360 | D02362 | D02408 |
| D02537 | D02574 | D02624 | D02716 | D03562 | D03740 |
| D03991 | D04048 | D04905 | D05077 | D06172 | D07283 |
| D07445 | D07447 | D07450 | D07473 | D07791 | D07845 |
| D07894 | D08098 | D08127 | D08174 | D08215 | D08283 |
| D08435 | D08458 | D08490 | D08690 | | |

**4. Drugs in target group "Enzymes"**

| | | | | | |
|---|---|---|---|---|---|
| D00107 | D00109 | D00112 | D00118 | D00120 | D00125 |
| D00126 | D00130 | D00131 | D00132 | D00141 | D00142 |
| D00145 | D00153 | D00160 | D00183 | D00184 | D00186 |
| D00196 | D00204 | D00210 | D00211 | D00212 | D00216 |
| D00217 | D00218 | D00220 | D00222 | D00224 | D00240 |
| D00251 | D00256 | D00257 | D00258 | D00259 | D00260 |
| D00262 | D00263 | D00264 | D00273 | D00296 | D00297 |
| D00298 | D00302 | D00310 | D00315 | D00317 | D00321 |
| D00322 | D00330 | D00333 | D00350 | D00351 | D00353 |
| D00355 | D00359 | D00362 | D00401 | D00410 | D00412 |
| D00413 | D00414 | D00416 | D00417 | D00421 | D00423 |
| D00424 | D00429 | D00434 | D00435 | D00445 | D00447 |
| D00448 | D00450 | D00453 | D00455 | D00459 | D00466 |
| D00469 | D00488 | D00497 | D00510 | D00518 | D00535 |
| D00558 | D00562 | D00564 | D00567 | D00574 | D00578 |
| D00579 | D00580 | D00582 | D00587 | D00592 | D00621 |
| D00622 | D00624 | D00652 | D00653 | D00667 | D00670 |
| D00707 | D00718 | D00724 | D00749 | D00753 | D00762 |
| D00781 | D00785 | D00786 | D00870 | D00871 | D00872 |
| D00873 | D00874 | D00878 | D00879 | D00880 | D00887 |
| D00889 | D00893 | D00894 | D00895 | D00896 | D00900 |
| D00902 | D00903 | D00904 | D00905 | D00906 | D00907 |
| D00908 | D00909 | D00910 | D00911 | D00912 | D00917 |
| D00919 | D00920 | D00921 | D00922 | D00923 | D00927 |
| D00929 | D00939 | D00960 | D00963 | D00964 | D00968 |
| D00970 | D00994 | D00995 | D01001 | D01049 | D01053 |
| D01055 | D01070 | D01074 | D01075 | D01093 | D01133 |
| D01136 | D01142 | D01147 | D01155 | D01157 | D01178 |
| D01180 | D01196 | D01199 | D01223 | D01240 | D01251 |
| D01276 | D01280 | D01283 | D01325 | D01334 | D01415 |
| D01489 | D01526 | D01560 | D01572 | D01581 | D01601 |
| D01628 | D01653 | D01687 | D01720 | D01739 | D01811 |
| D01819 | D01844 | D01896 | D01904 | D01949 | D01982 |

| | | | | | |
|---|---|---|---|---|---|
| D02085 | D02119 | D02121 | D02137 | D02166 | D02190 |
| D02193 | D02194 | D02196 | D02199 | D02201 | D02203 |
| D02216 | D02221 | D02222 | D02228 | D02229 | D02241 |
| D02258 | D02267 | D02299 | D02301 | D02306 | D02307 |
| D02318 | D02323 | D02336 | D02339 | D02344 | D02345 |
| D02348 | D02350 | D02353 | D02365 | D02368 | D02376 |
| D02405 | D02415 | D02418 | D02441 | D02471 | D02481 |
| D02482 | D02483 | D02495 | D02501 | D02555 | D02558 |
| D02709 | D02922 | D02923 | D03034 | D03082 | D03150 |
| D03208 | D03211 | D03349 | D03410 | D03428 | D03432 |
| D03546 | D03656 | D03710 | D03715 | D03731 | D03752 |
| D03753 | D03769 | D03771 | D03795 | D03798 | D03820 |
| D03822 | D03828 | D03882 | D04008 | D04112 | D04196 |
| D04197 | D04515 | D04720 | D05021 | D05022 | D05032 |
| D05177 | D05353 | D05407 | D05411 | D05529 | D05590 |
| D05947 | D06074 | D06412 | D06503 | D06637 | D06675 |
| D06881 | D07057 | D07064 | D07452 | D07471 | D07472 |
| D07474 | D07487 | D07596 | D07598 | D07614 | D07621 |
| D07626 | D07635 | D07636 | D07639 | D07643 | D07644 |
| D07645 | D07647 | D07648 | D07650 | D07651 | D07653 |
| D07654 | D07658 | D07659 | D07661 | D07698 | D07712 |
| D07733 | D07751 | D07782 | D07869 | D07871 | D07892 |
| D07896 | D07901 | D07925 | D07961 | D07965 | D07992 |
| D08011 | D08012 | D08109 | D08120 | D08143 | D08218 |
| D08237 | D08261 | D08306 | D08380 | D08401 | D08410 |
| D08469 | D08514 | D08534 | D08556 | D08557 | D08605 |
| D08618 | D08668 | | | | |
